# Burnout and Personality Profiles Among Chinese Nurses

**DOI:** 10.3390/bs14121117

**Published:** 2024-11-21

**Authors:** Sijun Zhang, Ke Xiao, Zhen Tian

**Affiliations:** 1 Institute of Educational Sciences, Hunan University, Changsha 410012, China; 2 Medical Management Department, Health Commission of Hebei Province, Shijiazhuang 050000, China; kexiaohebei@163.com; 3 School of Architecture and Planning, Hunan University, Changsha 410012, China; 4 Austria-China Low Carbon Building and Energy Joint Laboratory, Changsha 410012, China

**Keywords:** burnout, personality profile, nurse, demographics

## Abstract

Although many studies have examined the relationship between the five dimensions of personality and nurse burnout, few studies have examined the relationship between nurse burnout and the overall personality profile. In addition, nurses’ demographic characteristics have been found to be related to nurses’ burnout level, but the results have been inconsistent. This study aimed to determine personality profiles based on the Big Five personality model in a sample of Chinese nurses then analyze the relationship between burnout and personality profiles and demographics. A total of 1423 nurses were sampled and assessed using the Big Five Inventory and the Oldenburg Burnout Inventory. A k-means cluster analysis was used to divide participants into different personality profiles. Multivariate analysis of variance and binomial logistic regression were used to examine the relationship of burnout with personality profiles and demographics. Cluster analysis identified three personality profiles among nurses: resilient, ordinary, and distressed. For each dimension of the Big Five Inventory and Oldenburg Burnout Inventory, there was a significant difference between different personality profiles, with effect sizes being from 0.37 to 0.57. Nurses with a distressed personality profile were 4.52 times more likely to be diagnosed with burnout than nurses with an ordinary profile, while nurses with a resilient personality profile had a 55% reduction in burnout compared to nurses with an ordinary profile. The results suggested that hospital administrators should focus on nurses with a distressed personality profile to identify potential burnout nurses as early as possible. The findings also enabled hospital administrators to consider the different personality profiles of nurses and the specific requirements of the job to select suitable candidates.

## 1. Introduction

### 1.1. Literature Review

Maslach and Jackson defined burnout as a psychological syndrome with three dimensions: emotional exhaustion, depersonalization, and reduced perception of personal accomplishment [1]. Emotional exhaustion refers to feeling overextended and physically and emotionally depleted in the workplace, depersonalization refers to alienation from others (e.g., coworkers or patients), and reduced perception of personal accomplishment refers to feelings of incapability and doubt about one’s professional abilities [2].

Studies in different countries have identified nurses as a population at high risk of developing burnout [3,4,5,6]. Aiken et al. reported that 13–78% of nurses in Europe and the USA consider themselves to be burned out [5]; the situation is similar in China, where 59–69% of nurses are at a high risk of developing burnout and 7.4% are diagnosed as burned out [7,8,9]. Individual nurse burnout is associated with psychological distress, insomnia, lower job satisfaction, somatic complaints, and substance abuse [10,11,12,13], and burnout in nurses is communicable and can be passed from one nurse to another [14]. As a result, burnout is not only a problem for the individual nurse but for the whole hospital, as nurse burnout significantly increases costs due to inefficiency, quitting, and recruitment difficulties [5,14,15,16,17,18]. However, burnout affects not only the individual nurse and the hospital but also the patients. Nurses’ depersonalization and emotional exhaustion can affect patient satisfaction and safety [12,19,20,21,22,23], and nurse burnout leads to a reduction in compassion and caring, and patients perceive lower quality of care in such circumstances [24].

Although job-related factors (e.g., lack of clarity in feedback, work overload, hospital management, job complexity, and recurrent night work) are significant determinants of the development of nurse burnout [25,26,27,28], not all nurses who experience the same job-related factors develop burnout, suggesting the importance of individual factors in determining the occurrence of burnout. Among individual factors, many researchers have shown that personality traits can be considered as significant predictive factors for the development of nurse burnout [29,30,31,32]. The most commonly used personality model in nurse burnout studies is the Big Five personality traits. This model defines five groups of personality traits that are interdependent: neuroticism, extraversion, openness, agreeableness, and conscientiousness [33]. Iorga et al. and Grigorescu et al. found that neuroticism was associated with a higher level of burnout in nurses [29,34], while three studies found that conscientiousness was negatively related to burnout in nurses [35,36,37]. Iorga et al. and Chang et al. found that perfectionist nurses were not fully satisfied with the results of their work, and therefore, their exhaustion was significantly higher than that of other nurses [29,38]. Agreeableness and openness, like conscientiousness, have been inversely related to the development of burnout in nurses [29,39,40]. Although many studies have examined the relationship between the five dimensions of personality and nurse burnout, few studies have investigated the relationship between nurse burnout and overall personality type/profile. Traditionally, there are four personality types: A, B, C, and D. Type A personality is driven by a sense of urgency, high ambition, and a competitive temperament. Individuals with type B personality prefer to go with the flow and are flexible, relaxed, and internally satisfied. Individuals with type C personality are cautious, focused, and consistent in all aspects of their lives. Individuals with type D personality have a high tendency to experience negative emotions across time and situations and to escape when faced with challenges [41,42]. Two studies found that nurses with type D personality had less professional satisfaction and more job stress, which led them to develop more burnout [43,44], but no study investigated the relationship between nurse burnout and other personality types, and ABCD personality types lack a valid measurement tool [45]. Perez-Fuentes et al. found three personality profiles in nurses through cluster analysis and investigated the relationship between nurse burnout and personality profile [46]; however, this study used Big Five Inventory-10 (a short version of Big Five Inventory-44). Studies have shown that cluster analysis is inappropriate for the short version Big Five Inventory-10 [47,48].

Nurses’ demographic characteristics such as age, gender, job rank, academic degree, and experience have also been found to be associated with nurses’ burnout level, but the results have been inconsistent. For example, most studies reported that younger nurses tended to have higher burnout scores than older nurses [32,49,50,51,52,53,54], while a study in Nigeria and a study in Portugal found that older nurses had higher burnout scores [28,55]. Moreover, studies from Japan [52], Iran [21], and Nigeria [53] found that female nurses were more likely to experience burnout than their male counterparts, while another study in China [51] found the opposite. In addition, a study from China [50] suggested that senior nurses were more likely to experience burnout, while results from articles from Nigeria [28] and Hungary [56] suggested that junior nurses were more at risk.

### 1.2. Research Questions and Objectives

Previous studies focused on the relationship between the five dimensions of personality and nurse burnout, while the relationship between nurse burnout and the overall personality profile has not been thoroughly investigated. In practice, burnout prevention and intervention for a nurse is based on his/her overall personality profile, not a single personality dimension; therefore, focusing on personality profiles, as opposed to individual personality dimensions, is significant to burnout prevention and intervention. In addition, the relationship between nurses’ demographic characteristics and burnout has been found to be inconsistent in previous studies; this inconsistency might be due to the ignorance of personality, and the distribution of personality profiles between distinct nurse populations might be significantly different. This study aimed to identify personality profiles based on the Big Five personality model in a sample of Chinese nurses and then to analyze the relationship of burnout with personality profiles and demographics in this sample. The results of this study may help hospital administrators to implement burnout prevention and intervention that are appropriate for different groups of nurses with distinct personality profiles, thus enabling large-scale prevention and intervention of nurse burnout in hospitals.

## 2. Materials and Methods

### 2.1. Study Design

This study was quantitative, correlational, and cross-sectional. The data collection method used was a questionnaire.

### 2.2. Participants

A total of 1783 questionnaires were distributed, and 1665 were returned. Of the 1665 returned questionnaires, 242 were missing from the Big Five Inventory and Burnout Inventory, and finally, 1423 nurses were included in the statistical analysis. Of the 1423 nurses, 24 were male, 1388 were female, and 11 nurses were missing gender information; the average age of the nurses was 31.3 years old, and 16 nurses’ age information was missing; for educational level, 326 nurses had no college degree, 1083 held a bachelor’s degree and above, and 14 nurses’ educational information was missing; 669 nurses’ working time was less than five years, 718 nurses had worked for more than five years, and 36 nurses’ working time information was missing; for nurses’ titles, 437 were nurses, 640 were nurse practitioners, 292 were supervisory nurse practitioners, 30 were associate nurse practitioners and above, and 24 nurses’ title information was missing; for nurses’ positions, 147 were head nurses, 1223 were general nurses, and 53 nurses’ position information was missing.

### 2.3. Study Procedures

A multistage cluster sampling method was used in Hebei Province, China. In the first stage of sampling, all cities in Hebei Province were divided into four clusters according to their urbanization level, and within each cluster, all cities were numbered. A starting point was randomly selected from the random number table, a random number was drawn for each city in turn, and the random numbers obtained were arranged in ascending order. The city with the smallest random number in each cluster was selected as the sampling city, and four cities were finally identified: Tangshan, Langfang, Hengshui, and Zhangjiakou. In the second stage, the sampling frame was all tertiary hospitals in four cities, and the same random number table method was used to randomly select one tertiary hospital from each of the four cities. In the third stage, 1/2 of the departments of internal medicine, surgery, obstetrics, and pediatrics in each of the four tertiary hospitals were randomly selected, and all nurses in the selected departments were included in this study.

This study was approved by the Ethics Committee of Health Commission of Hebei Province on 7 March 2023. Approval to use the Chinese version of the Big Five Inventory and the Chinese version of the Oldenburg Burnout Inventory was obtained from Zhang [57] and Xu et al. [58] in May 2023. A total of 1783 nurses from tertiary hospitals were sampled in July 2023, and 1665 answered the questionnaire online in September and October 2023. The researchers conducted statistical analyses after October 2023.

### 2.4. Measures

#### 2.4.1. Big Five Inventory-44

The most commonly used personality measure in nurse burnout studies is the Big Five Inventory, the five dimensions of personality have been found to be associated with nurse burnout. To identify personality profiles based on the Big Five personality model via cluster analysis, Big Five Inventory-44 is recommended [47,48]. Big Five Inventory-44 is a 44-item 5-point Likert scale that assesses five dimensions of personality: neuroticism, extraversion, openness, agreeableness, and conscientiousness [33]. This study used the Chinese version, which has been widely used in previous studies; it was reported that reliability (as measured by Cronbach’s alpha) was 0.77 for the neuroticism scale, 0.79 for the extraversion scale, 0.83 for the openness scale, 0.81 for the agreeableness scale, and 0.71 for the conscientiousness scale [57].

#### 2.4.2. Oldenburg Burnout Inventory

The Oldenburg Burnout Inventory was developed by Demerouti and Nachreiner in 1998 and is a 16-item 4-point Likert scale that describes different states of exhaustion and disengagement [59]. Half of the 16 items are positively worded, while the others are negatively worded (scored in reverse order). Its theoretical model is based on the assumption that burnout is a two-dimensional syndrome that can occur regardless of one’s occupation. The validity of the Oldenburg Burnout Inventory has been tested in different populations [60,61,62,63]. The original version was proposed in German; this study used the Chinese version, which has been widely used by Chinese researchers, and it was reported that reliability (as measured by Cronbach’s alpha) was 0.93 for the exhaustion scale and 0.88 for the disengagement scale [58].

#### 2.4.3. Demographic Variables

Previous studies have shown that age, gender, experience and job rank are associated with nurses’ burnout level. As educational level, title, and position are related to nurses’ job rank in China, this part of the questionnaire included items about the participants’ gender, age, educational level, years of work, title, and position.

### 2.5. Statistical Analysis

Statistical analyses were carried out using R, version 4.3.2. The five dimensions of the Big Five Inventory were used as the clustering variables, correlation analyses and normality tests were used to check whether the data met the basic conditions of cluster analysis (i.e., weak correlation and normal distribution), and then the clustering variables were standardized. There are two common methods of cluster analysis: hierarchical clustering and k-means clustering. K-means is a partitioning algorithm that divides the data into a predefined number of clusters (k). It iteratively assigns each data point to the nearest cluster center and updates the cluster centers until convergence is reached. Hierarchical clustering builds a hierarchy of clusters either by iteratively merging small clusters or by dividing a large cluster. The output is a dendrogram that visualizes the hierarchical structure of clusters, so hierarchical clustering is computationally intensive, especially for large datasets [64]. Previous studies found that individuals with high neuroticism, low conscientiousness, and low extraversion are prone to distress [65,66,67,68]; therefore, this personality profile was referred to as the distressed profile; other studies in anesthesiologists [69] and nurses [46] identified another personality type that is the opposite of the distressed personality profile; this personality profile was referred to as the resilient personality. Moreover, Perez-Fuentes et al. found three personality profiles in nurses via cluster analysis [46]. Based on these previous studies, the number of clusters in this study was predefined as 2 or 3. In addition, 1423 participants were analyzed in this study; hierarchical clustering was inappropriate with such a large dataset, so a k-means cluster analysis was performed to divide the participants into different personality profiles. In order to find the optimal number of clusters for a k-means cluster analysis, we used the elbow method, the silhouette method, the gap statistic method, and consensus-based algorithm, with the help of package “parameters” and “ggplot2” in R.

Five dimensions of the Big Five Inventory and two dimensions of the Oldenburg Burnout Inventory were defined as dependent variables to test for differences between personality profiles. For each personality profile, the scores on five dimensions of the Big Five Inventory and two dimensions of the Oldenburg Burnout Inventory were normally distributed, so seven multivariate analyses of variance were conducted for between-personality profile comparisons.

The χ^2^ tests were used to compare the demographic characteristics of nurses with different personality profiles. Binomial logistic regression was used to examine the association between burnout and personality profile.

### 2.6. Ethical Considerations

This study was conducted in compliance with ethical standards and was approved by the Health Commission of Hebei Province Ethics Committee (No: 24577307). Informed consent was obtained from all participants. All the participants in this study were informed of the purpose of the study before being asked to answer the questionnaire. Moreover, autonomy to participate in the study was guaranteed, and information was kept confidential and used only for scientific research. Anonymity was ensured by using anonymous surveys, which cannot be traced back to the respondent. The survey did not contain any personally identifiable information such as names or contact information.

## 3. Results

### 3.1. Nurses’ Personality Profiles

The correlation coefficients between the dimensions of the Big Five Inventory ranged from −0.49 to 0.58, the scores of the five dimensions of the Big Five Inventory were normally distributed, and basic conditions for cluster analysis were met. For the optimal number of clusters for k-means cluster analysis, the elbow method suggested three clusters, the silhouette method suggested three clusters, the gap statistic method suggested four clusters, and the consensus-based algorithm suggested three clusters. Given that the number of clusters in this study was predefined as two or three, the optimal number of clusters was determined to be three.

**Figure 1:**
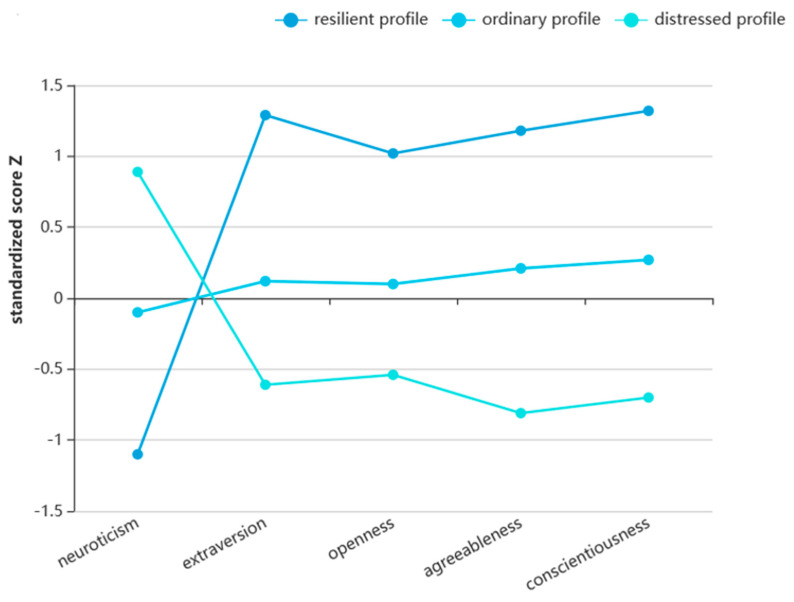
Cluster analysis results of nurses’ personality.

The results of the k-means cluster analysis showed that the nurses’ personality could be divided into three profiles (see Figure 1): (1) Resilient profile—249 nurses were categorized into this personality profile; they had low neuroticism, high extraversion, high openness, high agreeableness, and high conscientiousness, and the average score on neuroticism was below (mean − standard deviation), while the average scores on the other dimensions were above (mean + standard deviation). (2) Ordinary profile—601 nurses were categorized into this personality profile; they had medium neuroticism, extraversion, openness, agreeableness, and conscientiousness (average scores fell in (mean − 1/2 standard deviation, mean + 1/2 standard deviation)). (3) Distressed profile—569 nurses were categorized into this personality profile; they had high neuroticism, low extraversion, low openness, low agreeableness, and low conscientiousness.

Five MANOVAs showed that for each dimension of the Big Five Inventory, there was a significant difference between different personality profiles (see Table 1), with effect sizes (partial eta squared, η^2^) being large (0.37–0.57).

The χ^2^ test showed a significant difference in the distribution of personality profiles between head nurses and general nurses (χ^2^ = 10.74, *p* < 0.01, see Table 2). The post hoc test showed that the percentage of head nurses with a resilient personality profile was significantly higher than that of general nurses, and the percentage of head nurses with a distressed personality profile was significantly lower than that of general nurses.

### 3.2. Relationship of Burnout to Personality Profiles and Demographics

For each dimension of the Oldenburg Burnout Inventory, there was a significant difference between the different personality profiles (see Table 1). Post hoc tests revealed that nurses with a resilient personality profile had the lowest scores on two dimensions of the Oldenburg Burnout Inventory, whereas nurses with a distressed personality profile had the highest scores on two dimensions.

According to Demerouti et al. (2001) and Xu et al. (2022), exhaustion was considered high if the score was greater than 23, and disengagement was considered high if the score was greater than 21. An individual whose scores in two dimensions of the Oldenburg Burnout Inventory met these standards could be diagnosed with burnout [58,61]. A total of 288 nurses (20.2%) in this study were diagnosed with burnout. A binomial logistic regression was conducted with the presence of burnout as the dependent variable and personality profiles and demographic variables as independent variables; the results are shown in Table 3. Compared to nurses with an ordinary personality profile, nurses with a distressed personality profile were 4.52 times more likely to be diagnosed with burnout, while nurses with a resilient personality profile had a 55% reduction in burnout. In addition, general nurses were 1.72 times more likely to be diagnosed with burnout than head nurses; male nurses were 3.16 times more likely to be diagnosed with burnout than female nurses, but as there were only 24 males in this study, we could not conclude that male nurses were more susceptible to burnout.

## 4. Discussion

### 4.1. Nurses’ Personality Profiles

In this study, three personality profiles were obtained in the group of nurses through cluster analysis, and there was a high degree of heterogeneity among the different personality profiles. Nurses with one personality profile had higher scores on neuroticism and lower scores on the other four dimensions. Previous studies found that individuals with high neuroticism, low conscientiousness, and low extraversion were prone to distress [65,66,67,68]; therefore, this personality profile was referred to as the distressed profile.

Previous studies in anesthesiologists [69] and nurses [46] identified another personality type that was the opposite of the distressed personality profile, with below-average scores on neuroticism and above-average scores on the other four dimensions; this personality type was termed the resilient personality. Researchers argued that individuals with a resilient personality could cope with stress, conflict, and uncertainty in the work environment [69,70]. Unlike these previous studies, this study identified two other personality profiles in addition to the distressed profile. Nurses with one of the personality profiles scored within (mean − 1/2 standard deviation, mean + 1/2 standard deviation) of the five dimensions, and we called this profile the ordinary profile. The other personality profile was characterized by low neuroticism, high conscientiousness, high extraversion, high agreeableness, and high openness, and its average score on neuroticism was below (mean − standard deviation), while its average scores on the other dimensions were above (mean + standard deviation). Previous studies have shown that openness, extraversion, agreeableness, and conscientiousness are positively correlated with an individual’s level of resilience, whereas neuroticism is negatively correlated with the level of resilience [26,70,71,72,73], so we have named this personality profile the resilient profile. “Resilience” is a stable and unique form of response formed by individuals in the process of coping with stress, which can help individuals buffer stress and maintain physical and mental health [26,71].

This study also found that the percentage of head nurses with a resilient personality profile was significantly higher than that of general nurses, which suggested that nurses with a resilient personality profile were more compatible with their job and had a higher likelihood of being promoted to head nurse. The difference in the distribution of personality profiles between head nurses and general nurses may explain the difference between this study and previous studies, and the possible reason is that this study included not only general nurses but also head nurses, thus distinguishing the ordinary profile from the resilient profile.

### 4.2. Relationship Between Personality Profiles and Nurse Burnout

This study showed that nurses with a resilient personality profile had the lowest scores on two dimensions of the Oldenburg Burnout Inventory, whereas nurses with a distressed personality profile had the highest scores on two dimensions of the Oldenburg Burnout Inventory. Previous studies found that conscientiousness and agreeableness were positively correlated with burnout, whereas neuroticism was negatively correlated with burnout [29,34,35,36,37,39,40,41]; these findings were consistent with the results of the current study. The results of the binomial logistic regression showed that nurses with a distressed personality profile were highly susceptible to burnout, the possible reason being that these nurses were more emotionally unstable, and they were not well able to resolve the psychological distress caused by work pressure, which made them susceptible to burnout. Nurses with a resilient personality profile had a significantly lower risk of burnout compared to nurses with other personality profiles, suggesting that the resilient personality profile may serve as a protective factor for individual development, enabling individuals to better cope with and regulate work stress.

Although previous studies examined the relationship between personality dimensions and burnout, they ignored the correlation between personality dimensions and failed to reflect the relationship between the combination of individual personality dimensions and burnout. Some researchers have argued that using a combination of the five dimensions of the Big Five Personality may better explain behaviors than using a single personality dimension [74]. In this study, nurses were divided into three personality profiles, which not only made it possible to examine the effects of different personality profiles on nurse burnout but also helped hospital administrators to implement interventions that are compatible with the nurses of different personality profiles and provided a reference for the implementation of personalized management.

### 4.3. Implications

This study suggests that hospital administrators should assess nurses using the Big Five Inventory to screen for nurses with a distressed personality profile in order to identify potential burnout nurses as early as possible. Furthermore, prevention and intervention for nurse burnout should be implemented according to personality profiles. For example, for nurses with a distressed personality profile, prevention is needed before burnout occurs. In addition, hospital administrators can consider nurses’ personality profiles and the specific job requirements to select suitable candidates, which provides an opportunity to optimize human resources in the hospital, maximize individual potential, and improve nurses’ performance and job satisfaction. For example, in order to prevent nurse burnout, hospital administrators should select nurses with a resilient personality profile to work in emergency departments.

### 4.4. Limitations

This study was conducted in one province in China and only included nurses from tertiary hospitals. Future studies could include nurses from more regions and different levels of hospitals to explore whether the three personality profiles in this study are universal in the nurse population. In addition, only 24 male nurses were recruited in this study, and the findings were for female nurses. This study also found a gender difference in the prevalence of burnout, which cannot be generalized to other nurse populations due to the small number of male nurses. Future studies could recruit more male participants to investigate the existence of a gender difference in nurse burnout. Furthermore, this study used a self-reported inventory to diagnose burnout, which may lead to misdiagnosis. Future studies could combine the Oldenburg Burnout Inventory with some physical symptoms, such as body aches, recurring headaches, and insomnia, to diagnose burnout more accurately.

## 5. Conclusions

This study used cluster analysis to identify three different personality profiles in a representative group of nurses in Hebei Province, China, and named them the resilient, ordinary, and distressed profiles. Nurses with a distressed personality profile were highly susceptible to burnout, whereas nurses with a resilient personality profile had a significantly lower risk of burnout. This study suggested that hospital administrators should focus on nurses with a distressed personality profile to identify potential burnout nurses as early as possible. Moreover, the study enabled hospital administrators to consider nurses’ personality profiles and the specific job requirements to select suitable candidates. Future studies should focus on the prevention of nurse burnout and the development of interventions based on different personality profiles. Further studies in different regions or health care settings are needed to test the generalizability of the three personality profiles and the relationship between burnout and personality profiles.

## Figures and Tables

**Table 1:** Comparisons of personality and burnout scores between different personality profiles.

Dimension	All Participants	Resilient Profile ①	Ordinary Profile ②	Distressed Profile ③	*F* Value	Post Hoc Test	*η* ^2^
Neuroticism	21.6 ± 4.4	16.0 ± 3.5	21.2 ± 2.6	24.9 ± 3.1	697.27 ***	②③ > ①, ③ > ②	0.50
Extraversion	25.2 ± 4.3	32.9 ± 3.1	25.8 ± 2.9	22.4 ± 3.2	436.18 ***	① > ②③, ③ > ②	0.44
Openness	3.17 ± 4.6	36.3 ± 4.5	32.4 ± 3.9	29.0 ± 3.9	268.55 ***	① > ②③, ② > ③	0.37
Agreeableness	34.6 ± 4.2	40.1 ± 3.0	35.8 ± 2.9	31.0 ± 3.3	799.46 ***	① > ②③, ② > ③	0.57
Conscientiousness	31.0 ± 4.0	37.1 ± 3.4	32.2 ± 3.2	27.8 ± 2.9	574.19 ***	① > ②③,② > ③	0.46
Exhaustion	19.1 ± 2.7	16.0 ± 2.0	18.9 ± 2.2	22.5 ± 2.1	515.49 ***	②③ > ①, ③ > ②	0.44
Disengagement	19.9 ± 3.0	16.2 ± 2.3	20.0 ± 2.1	23.7 ± 1.9	442.68 ***	②③ > ①, ③ > ②	0.41

*** denotes *p* < 0.001. For neuroticism, extraversion, openness, agreeableness, conscientiousness, exhaustion, and disengagement, the mean ± standard deviation is displayed.

**Table 2:** Comparison of personality profile distribution among nurses with different demographics.

Demographic Variable	Resilient Profile (*n* = 273)	Ordinary Profile (*n* = 643)	Distressed Profile (*n* = 620)	*χ* ^2^
Age				4.17
≤25	53 (19.3%)	116 (42.2%)	106 (38.5%)	
26–35	138 (16.2%)	348 (40.9%)	364 (42.9%)	
36–45	41 (17.6%)	112 (48.1%)	80 (34.3%)	
>45	9 (19.1%)	22 (46.8%)	16 (34.0%)	
Gender				2.33
Male	7 (29.2%)	11 (45.8%)	6 (25.0%)	
Female	244 (17.6%)	582 (41.9%)	558 (40.5%)	
Educational Level				1.80
No College Degree	57 (17.5%)	130 (39.9%)	139 (42.6%)	
Bachelor’s Degree and Above	188 (17.4%)	464 (42.8%)	431 (39.8%)	
Working Time				2.23
<5 years	122 (18.2%)	293 (43.8%)	254 (38.0%)	
>5 years	118 (16.4%)	297 (41.4%)	303 (42.2%)	
Title				5.97
Nurse	78 (17.8%)	188 (43.0%)	171 (39.1%)	
Nurse Practitioner	113 (17.7%)	256 (40.0%)	271 (42.3%)	
Supervisory Nurse Practitioner	40 (13.7%)	135 (46.2%)	117 (40.1%)	
Associate Nurse Practitioner and Above	10 (33.3%)	15 (50.0%)	5 (16.7%)	
Position				10.74 **
Head Nurse	38 (25.9%)	67 (45.6%)	42 (28.6%)	
General Nurse	203 (16.6%)	511 (41.8%)	509 (41.6%)	

** denotes *p* < 0.01. Count (percentage) is displayed.

**Table 3:** Results of the binomial logistic regression.

Independent Variable	*B*	SE	Wald	Odds Ratio [95% CI]
Position				
Head Nurse (General Nurse)	−0.57	0.21	7.35 **	0.58 [0.40, 0.86]
Gender				
Female (Male)	−1.19	0.47	5.40 *	0.32 [0.13, 0.80]
Personality Profile				
Distressed Profile (Ordinary Profile)	1.51	0.13	132.07 ***	4.52 [3.57, 5.93]
Resilient Profile (Ordinary Profile)	−0.86	0.19	19.97 ***	0.45 [0.31, 0.62]

* denotes *p* < 0.05; ** denotes *p* < 0.01; *** denotes *p* < 0.001. For the independent variable, the reference group was displayed in parentheses.

## Data Availability

The data presented in this study are available on request from the corresponding authors due to privacy.
